# Transepidermal water loss increases during murine food anaphylaxis and reflects reaction severity

**DOI:** 10.3389/fimmu.2025.1667569

**Published:** 2025-10-02

**Authors:** Benjamin N. Woerner, Joseph Abbo, Allison Wang, Melanie K. Donahue, Judy Hines, Jessica O’Konek, Simon P. Hogan, James R. Baker, Charles F. Schuler

**Affiliations:** ^1^ Mary H. Weiser Food Allergy Center, University of Michigan, Ann Arbor, MI, United States; ^2^ Graduate Program in Immunology, University of Michigan, Ann Arbor, MI, United States; ^3^ Division of Allergy and Clinical Immunology, Department of Internal Medicine, University of Michigan, Ann Arbor, MI, United States; ^4^ Michigan Nanotechnology Institute for Medicine and Biological Sciences, University of Michigan, Ann Arbor, MI, United States; ^5^ Department of Pathology, University of Michigan, Ann Arbor, MI, United States

**Keywords:** food allergy, anaphylaxis, transepidermal water loss (TEWL), food anaphylaxis, biomarker

## Abstract

**Background:**

An increase in transepidermal water loss (TEWL) presages food anaphylaxis in allergic humans during oral food challenges. We sought to determine whether similar TEWL changes occur in mouse food anaphylaxis models.

**Methods:**

Using a Tewameter™ Nano, a mouse-compatible device, TEWL measurements were conducted on the ear, paw, and abdomen of BALB/c mice. Because of the highest measurement reproducibility, the ear was selected for use in the study. Baseline TEWL measurements under varied conditions were evaluated. Histamine injections were given to evaluate a non-IgE-mediated reaction. Two IgE-based models of food anaphylaxis were utilized: (1) passive systemic anaphylaxis (PSA) with dinitrophenyl (DNP)-IgE sensitization and DNP-albumin challenge, and (2) active systemic anaphylaxis (ASA) with ovalbumin-alum immunization followed by ovalbumin challenges. Core temperature, reaction severity score, diarrhea, and TEWL were recorded. MCPT-1 was measured as a mast cell activation correlate.

**Results:**

TEWL was reproducibly measured on the ear (17.7 g/m^2^/h) and showed no baseline differences with time, sex, device used, oral gavage, or intravenous injection. TEWL increased during histamine (5.73 g/m^2^/h), PSA (3.46 g/m^2^/h), and ASA (3.61 g/m^2^/h) challenges. TEWL correlated with reaction severity across conditions and with core temperature change in PSA and ASA challenges. TEWL increased significantly for all models, whereas other markers such as reaction severity and temperature change varied by model utilized.

**Conclusion:**

TEWL is reliably measured on the mouse ear. TEWL increased under varied reaction conditions, and the stimulus used did not alter results. TEWL offers a novel, real-time, objective, and noninvasive measure of murine food anaphylaxis that corresponds to human pathophysiology.

## Introduction

Food allergy (FA) in the United States is an increasingly prevalent illness affecting nearly 8% of children and 10% of adults (1–3). Food anaphylaxis is the severe and sometimes fatal outcome of food allergen exposure and is responsible for a high healthcare burden (4–8). FA diagnosis remains problematic given that traditional testing methods, such as food-specific skin and blood IgE testing, provide poor positive predictive values and fail to predict severity or threshold of reactivity (9–11). The oral food challenge (OFC) remains the criterion standard for the diagnosis of FA despite the inherent risk of anaphylaxis (6, 7, 12). Clinical diagnosis is required to identify the anaphylaxis endpoint and relies entirely upon physician observation since there is no approved measuring device, which increases costs, uncertainty, and perceived risk (9, 13). Early diagnosis and subsequent treatment of anaphylaxis can reduce reaction severity and symptoms (14).

Transepidermal water loss (TEWL) is a well-established measure of net skin barrier permeability that has been used in the assessment of dermatological conditions and medications. TEWL is measured by using skin contact probes that are painless and noninvasive and can give real-time feedback continuously over multiple hours (15, 16). Our group has previously shown that an increase in TEWL precedes food anaphylaxis during clinical OFCs and may provide advanced warning for anaphylaxis (17). TEWL has yet to be evaluated in murine models of food anaphylaxis, and prior studies offer varied methods for TEWL measurement in mice (18–22). The sole existing objective measure for food anaphylaxis in murine models is rectal temperature, which is invasive and carries a risk of perforation (23). A noninvasive measure, such as TEWL, would be a useful alternative for defining murine anaphylaxis severity given the key role of murine models as pre-clinical models in defining mechanisms, diagnostics, and therapeutics in food anaphylaxis.

In the present study, we aimed to determine an optimal approach for TEWL measurement in the context of murine food anaphylaxis and then define whether TEWL could provide a useful cutaneous measurement as in human food anaphylaxis to support future mechanistic studies in this disease context.

## Materials and methods

### Animals

BALB/c mice were housed under standard pathogen-free conditions in a temperature- and humidity-controlled room with food and water provided. Euthanasia procedures were conducted in accordance with University of Michigan’s Unit for Laboratory Animal Management policies. Carbon dioxide at 30%–70% flow rate of chamber volume per minute was used as a primary method, and terminal bleeding was used as a secondary method.

Sex as a biological variable: Both male and female mice between the ages of 4 and 6 weeks were employed in this study. We did not observe differences between male and female mice.

Study approval: All procedures were approved by the Institutional Animal Care and Use Committee (IACUC) at the University of Michigan under PRO00011478.

### TEWL measurement

**Figure 1:**
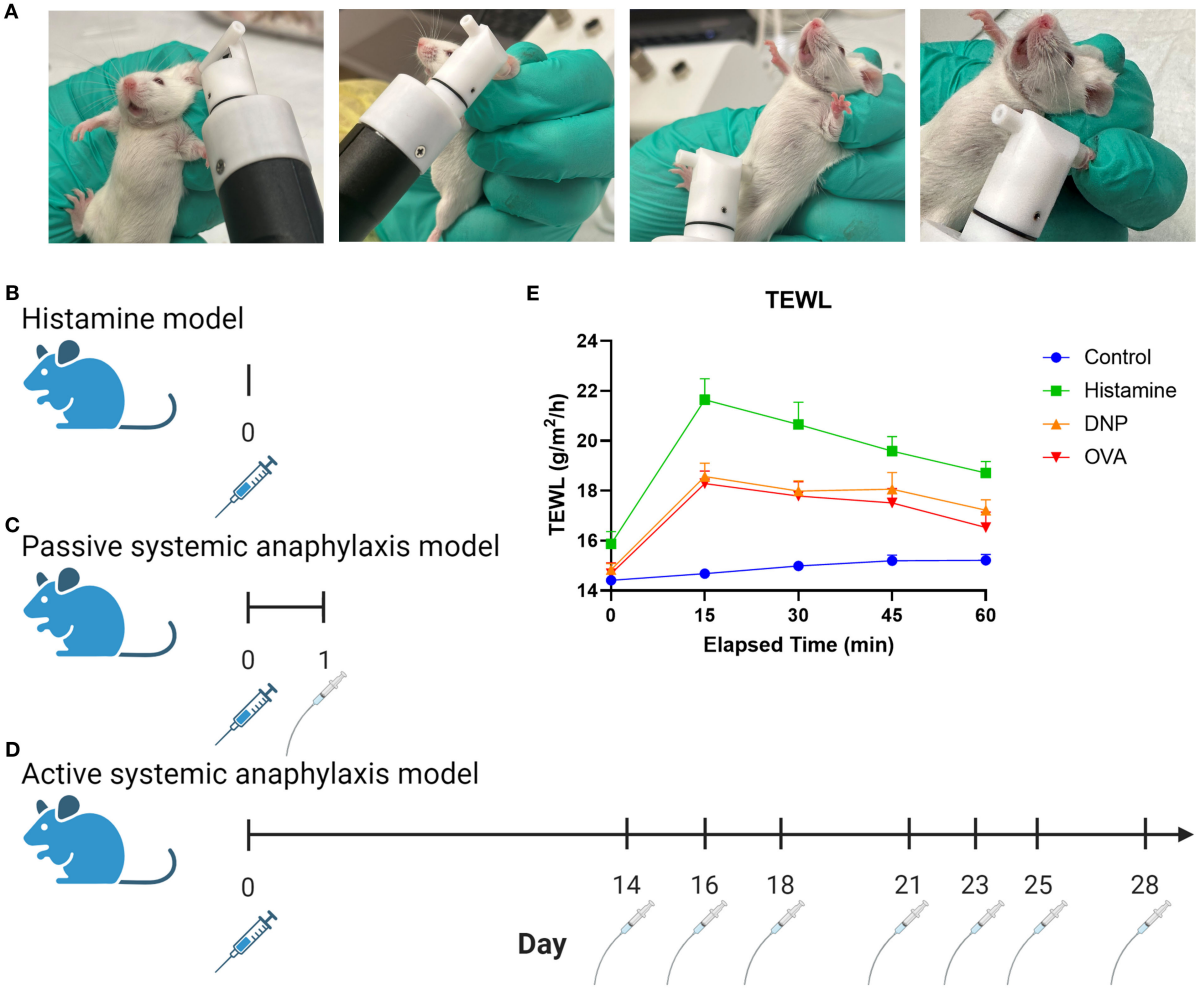
Graphic depiction of TEWL measurement and experimental models. **(A)** Images of tewameter placement on the murine ear, belly, and paw. **(B)** Non-IgE-mediated murine procedure using histamine via IV injection. **(C)** Murine PSA procedure using DNP sensitization via IV injection followed by oral gavage. **(D)** Murine ASA procedure using OVA sensitization with alum via IP injection followed by oral gavage challenges. **(E)** Measurement outcomes: anaphylaxis severity scoring, temperature, temperature change, TEWL and TEWL change, and MCPT-1.

TEWL was taken at baseline and at 15-min intervals throughout each experiment. TEWL was measured with a Tewameter™ Nano (Courage + Khazaka gmbh, Germany) placed on the surface of the skin. After an equilibration time of 25–30 s, five sets of 5-s measurements were captured. The paired software (MPA plus, Courage + Khazaka) was used to analyze each set of measurements and compute one mean value. TEWL was taken at the ear, paw, and abdomen of mice to find the most consistent place to measure (**Figure 1A**). Once the ear was selected as the location for all future measurements, captured by pinning between the finger and the tewameter (**Figure 1A**), all subsequent data represent an ear measurement unless otherwise specified.

### Histamine model

The mice were heated under a heat lamp (Model HL-1B 120v; Braintree Scientific) for 10 min before tail intravenous (IV) injection. The experimental mice received an IV injection in the tail with a volume of 50 μL per mouse of histamine (Fisher Scientific, AAL0919814) in phosphate-buffered saline (PBS; Cytiva HyClone, SH30256.01) at a concentration of 200 mg/mL (**Figure 1B**). Control mice received an injection of 50 μL of PBS. Physiological responses of TEWL, temperature, and reaction score were taken at baseline and at 15-min intervals for 60 min. After the trial, the mice were anesthetized with isoflurane (ULAM) and blood samples were collected for MCPT-1 enzyme-linked immunosorbent assay (ELISA) prior to euthanasia.

### Passive systemic anaphylaxis

All mice were sensitized with IV injections in the tail vein with 200 μL/100 μg per mouse of anti-dinitrophenyl (DNP)-IgE (Millipore Sigma, D8406) and PBS at a concentration of 50 μL/mL (**Figure 1C**). Prior to each challenge, all mice were starved for 5 h before experimental mice were challenged via oral gavage with a 50 mg/kg of DNP-albumin (Sigma-Aldrich, A6661) dissolved in 250 μL of PBS. The control mice were gavaged with 250 μL of PBS per mouse. Physiological responses of TEWL, temperature, and reaction score were taken at baseline and at 15-min intervals for 60 min. After the trial, the mice were anesthetized with isoflurane (ULAM) and blood samples were collected for MCPT-1 ELISA prior to euthanasia.

### Active systemic anaphylaxis

All mice were sensitized to ovalbumin (OVA) via intraperitoneal injection with a solution of 50 μg of OVA (Sigma-Aldrich, 9006-59-1) and 100 μL/kg of alum (InvivoGen, 21645-51-2) into 250 μL of PBS per mouse (**Figure 1D**). Two weeks following sensitization, the mice were challenged every 2–3 days for a total of seven challenges (23, 24). Prior to each challenge, all mice were starved for 5 h before experimental mice were orally gavaged 50 mg/kg of OVA dissolved in 250 μL of PBS, and the control mice were gavaged with 250 μL of PBS. Physiological responses of TEWL, temperature, and reaction score were taken at baseline and every 15 min for 60 min. After the trial, the mice were anesthetized with isoflurane (ULAM) and blood samples were collected for MCPT-1 ELISA prior to euthanasia.

### Additional physiological measurements

Core temperature was measured via a lubricated rectal probe (Model RET-3; Physitemp Instruments Inc.) following 5-s equilibration after insertion (Model Bat-12; Physitemp Instruments Inc). Reaction score was judged at each 15-min interval. The mice were assigned a score of 1 through 5 according to standard scoring, where 1 = excessive itching, 2 = hunching, 3 = labored breathing, 4 = moribund, and 5 = death (**Figure 1E**) (23). Diarrhea score is recorded binarily with 0 meaning no diarrhea occurred and 1 meaning it has.

Baseline TEWL was taken under various experimental control conditions. Mice were heated under a heat lamp (Model HL-1B 120v; Braintree Scientific) for either 5, 10, or 15 min and their TEWL and temperature were recorded every 15 min for 60 min. The mice were also placed under isoflurane until they were unconscious. The mice had their TEWL and temperature taken every 15 min for 60 min. Tail vein IV injections via a 26G ½ inch needle (Exel International, 14-841-32) and intragastric gavage via a reusable feeding needle (GloMed Inc., NC1299558) were also evaluated for their effect on baseline TEWL.

### MCPT-1 ELISA

Following the last anaphylaxis event of each trial, blood was obtained from each mouse via cardiac puncture. The blood was placed into an ice bucket for 30 min before centrifugation at 9,000 rpm for 10 min to collect serum. The serum was stored at −80 °C until analysis. MCPT-1 levels were quantified using the Mouse MCPT-1 (mMCP-1) Uncoated ELISA kit (Invitrogen, 88-7503) according to the manufacturer’s instructions. At the end of the procedure, once the stop solution was added, the plate was read at 450 nm using the GloMax® Explorer plate reader (Promega, GM3500) and concentrations were calculated using a standard curve.

### Statistical analysis

All statistical analyses were performed using GraphPad Prism (GraphPad Software, 10.4.1). Data were assessed for normality using the Shapiro–Wilk test. Comparisons between two groups were conducted using unpaired two-tailed *t*-tests for normally distributed data or the Mann–Whitney *U* test for non-normally distributed data. For comparisons involving more than two groups, one-way analysis of variance (ANOVA) followed by Tukey’s Honestly Significant Difference test was used. For the area under the curve (AUC) analysis, we calculated the area of the TEWL results above the baseline set for the food challenge by measurement at time 0 for each mouse. Where relevant, simple linear regressions were fit to *XY* data with an *R* ^2^ and *p*-value reported. Data are presented as mean ±standard error of the mean (SEM) or median with interquartile range (IQR) as appropriate. Statistical significance was defined as *p*<0.05.

## Results

### Baseline TEWL measurements

**Figure 2:**
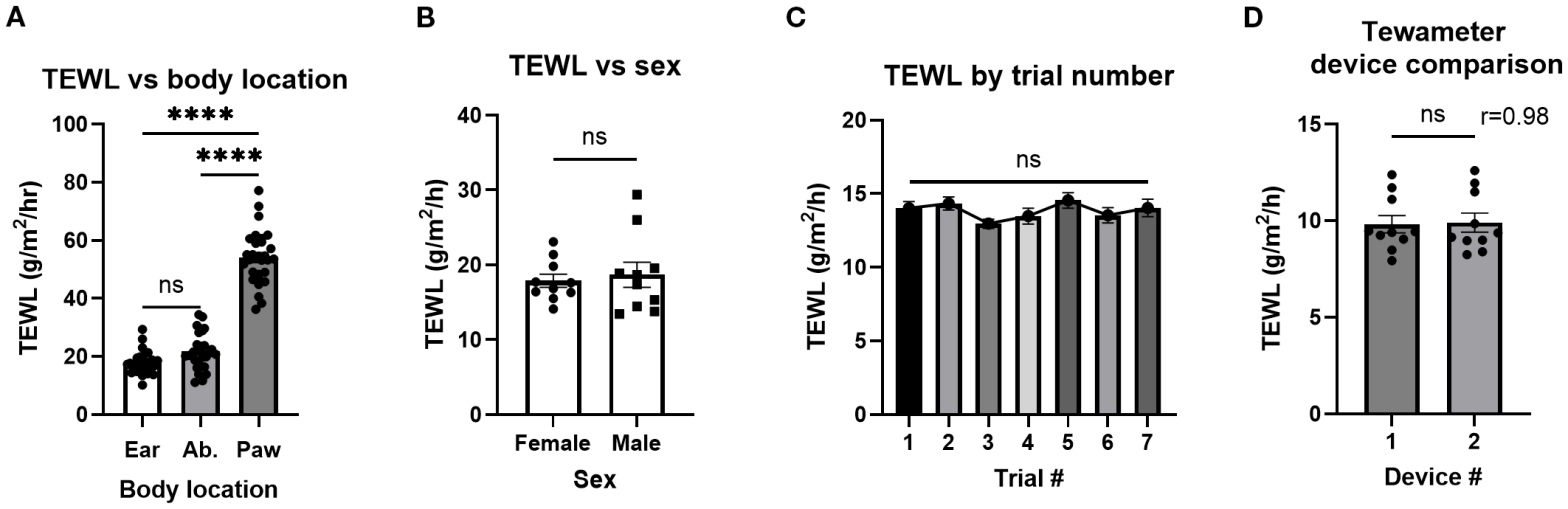
TEWL of different murine body parts, sexes, and a tewameter device comparison. **(A)** TEWL measurements of mouse ear, abdomen(Ab.), and paw; *n* = 30 per group. Ordinary one-way ANOVA. **(B)** TEWL measurements of male and female mice; *n* = 10 per group, unpaired *t*-test. **(C)** Baseline TEWL measurements of control mice for the seven trials making up the experiment. Trials 1–5, *n* = 24; trial 6, *n* = 17; trial 7, *n* = 11, mixed-effects analysis. **(D)** TEWL measurements using both available tewameters, *n* = 10, paired *t*-test. ****p<0.0001. ns, not significant.

To determine which part of the murine body would have TEWL results most reflective of human physiology (17), three body parts were tested: ear (mean, 17.7 g/m^2^/h), abdomen (mean, 21.8 g/m^2^/h), and paw (mean, 54.1 g/m^2^/h) (**Figure 2A**). The ear was most similar in TEWL to the human volar forearm data taken from OFCs as previously reported by our group (17) and was convenient to take measurements from. The abdomen posed the risk of the mouse urinating on the instrument and was not viable for repeated measures. Paw TEWL values were significantly greater than the ear and abdomen TEWL values (**Figure 2A**). All TEWL measurements aside from those explicitly labeled with a body site (**Figure 2A**) were conducted using the ear only.

To establish whether sex impacted TEWL measurement, the TEWL of both male (mean, 18.7 g/m^2^/h) and female (mean, 17.9 g/m^2^)/h) mice was compared with no significant differences observed (**Figure 2B**). TEWL from control mice over the course of seven non-reactive food challenges over a 17-day period [akin to the repeated reactions used in the active systemic anaphylaxis (ASA) studies later] was examined to determine that TEWL values were stable over time with no significant differences (**Figure 2C**). To confirm that both tewameters report similar measurements, a device comparison showed that minimal variation between tewameters was conducted (means, 9.8 and 9.9 g/m^2^/h) (**Figures 2D, E**).

### Control TEWL measurements

**Figure 3:**
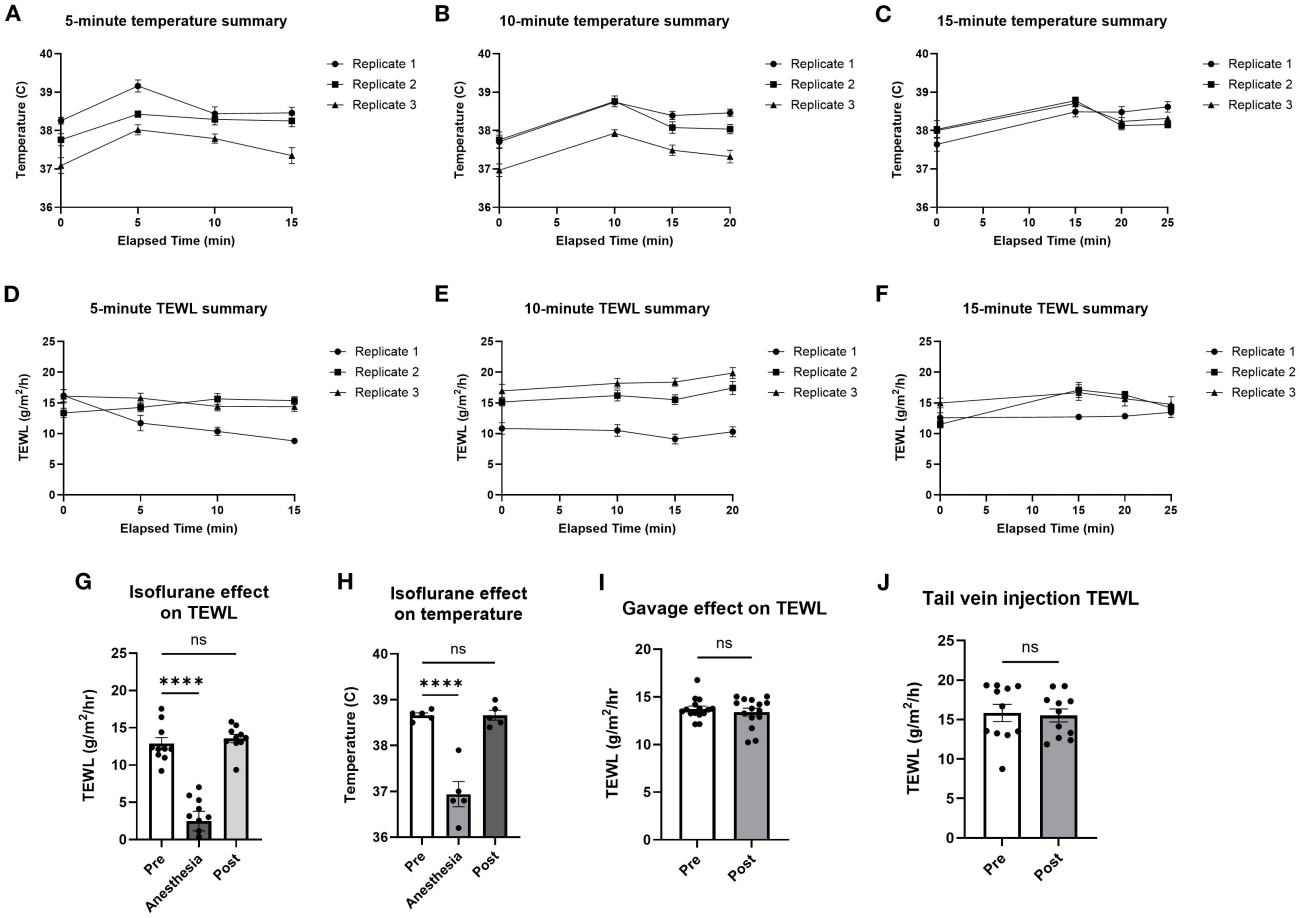
TEWL and temperature of mice before and after heat lamp trials, isoflurane trials, and oral gavage trials. Core temperature of mice was taken at baseline before being warmed for 5 min **(A, D)**, 10 min **(B, E)**, and 15 min **(C, F)** and in 5-min intervals after being warmed under the heat lamp; *n* = 10 per group. **(G)** Core temperature of mice taken at baseline, during, and after being induced with isoflurane anesthesia; *n* = 5 per group, * indicates statistical significance. **(H)** TEWL of mice taken at baseline, during, and after being induced with isoflurane anesthesia; *n* = 10 per group. **(I)** TEWL of mice taken at baseline and after being orally gavaged with saline; *n* = 15 per group. **(J)** TEWL of mice taken at baseline and after tail IV injection of saline; *n* = 11 per group. ****p<0.0001, ns, not significant.

To identify the impact on TEWL of the heat lamp used during tail vein IV injections, mice were warmed under the heat lamp for 5, 10, and 15 min before core temperature and TEWL were taken in parallel at 5-min intervals for 15 min after warming (**Figures 3A–F**). While core temperature increased after heating, TEWL did not vary significantly.

To analyze the effect of isoflurane anesthesia, TEWL and temperature measurements were taken before (mean = 38.7 °C; 12.9 g/m^2^/h), during (mean = 36.9 °C; 2.5 g/m^2^/h), and after (mean = 38.7 °C; 13.6 g/m^2^/h) anesthesia (**Figures 3G, H**). Time from anesthesia onset to TEWL measurement was 2 min. To measure the effect of oral gavage on TEWL, TEWL was taken before (mean = 13.7 g/m^2^/h) and after (mean = 13.4 g/m^2^/h) oral gavage of PBS (**Figure 3I**). To identify the effect of tail IV injection on TEWL, TEWL was taken before (mean = 15.8 g/m^2^/h) and after (mean = 15.5 g/m^2^/h) tail IV injection of PBS (**Figure 3J**). Time from gavage or injection to TEWL measurement was 1 min. Overall, anesthesia clearly impacted TEWL measurements, so anesthesia was only used after challenge for terminal bleeding to avoid affecting TEWL measurements during anaphylaxis. Heating, gavage, and IV injections appeared to have no significant effect on TEWL and so were used in subsequent anaphylaxis models.

### Non-IgE-mediated anaphylaxis—histamine

**Figure 4:**
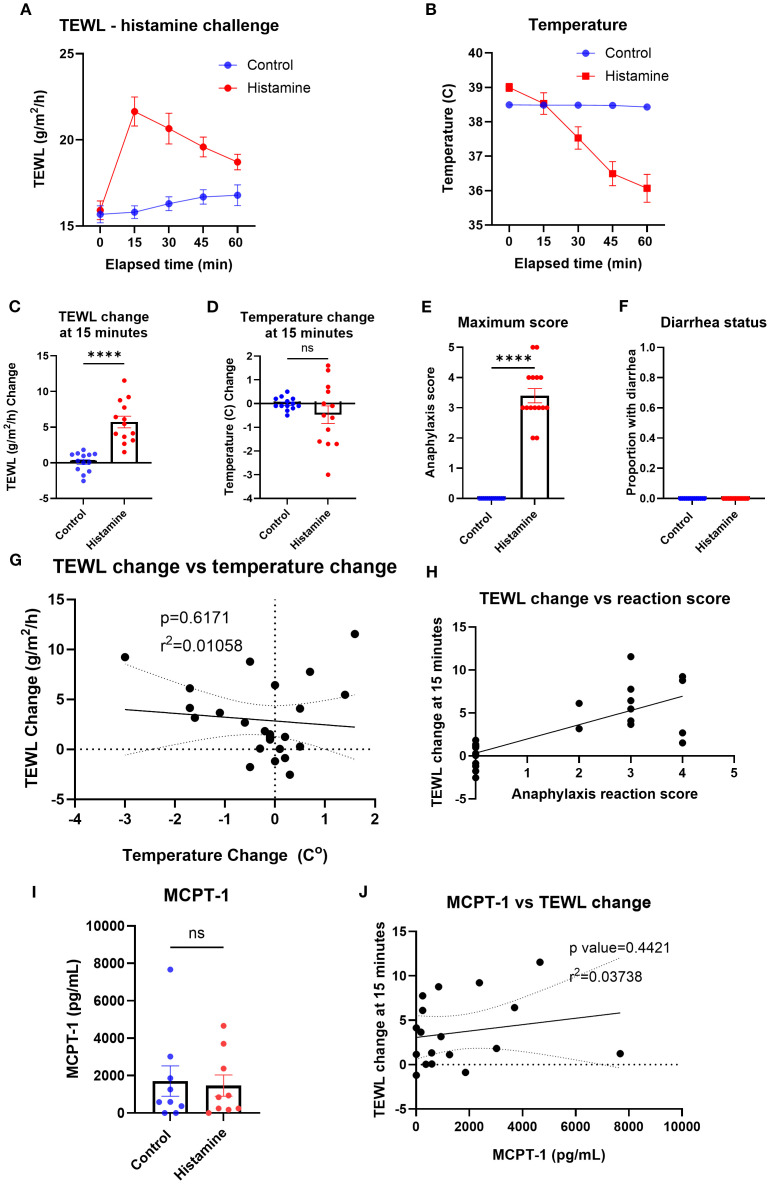
TEWL, temperature, anaphylaxis score, diarrhea status, and MCPT-1 measurements of control mice and mice administered histamine. **(A)** TEWL of mice taken at baseline and in 15-min intervals post-challenge for 60 min, *n* = 13 per group. **(B)** Internal temperature of mice taken at baseline and in 15-min intervals post-challenge for 60 min, *n* = 13 per group. **(C)** TEWL of mice at 15 min subtracted from baseline TEWL to get TEWL change at 15 min. *n* = 13 per group, unpaired *t*-test. **(D)** Internal temperature of mice at 15 min subtracted from baseline TEWL to get TEWL change at 15 min. *n* = 13 per group, unpaired *t*-test. **(E)** Highest score given to each mouse throughout the trial; *n* = 13 for each group; unpaired *t*-test. **(F)** Diarrhea status given to each mouse throughout the trial. *n* = 13 for each group; unpaired *t*-test. **(G)** Correlation of the mice’s TEWL change and their temperature change at 15 min. *n* = 26, simple linear regression. **(H)** Correlation of the mice’s TEWL change with their reaction score. *n* = 26 per group, ordinary one-way ANOVA. **(I)** Interpolation of MCPT-1 concentration found in the serum of control and histamine mice, *n* = 9 per group, unpaired *t*-test. **(J)** Interpolation of MCPT-1 concentration of murine serum correlated with TEWL change at 15 min; *n* = 9 per group, nonlinear fit. ****p<0.0001, ns, not significant.

To identify the impact of a non-IgE-mediated anaphylaxis-like event on the TEWL of mice, the TEWL and temperature of the control and histamine groups were measured at baseline and in 15-min intervals after challenge with histamine (**Figures 4A, B**). The greatest change in TEWL occurred at the 15-min time point (mean control = 0.12 g/m /h; histamine = 5.7 g/m^2^/h), while temperature decreased more slowly and was minimally different at 15 min (mean control = −0.01°C; histamine = −0.47°C) and continued to decline at a significant rate for the duration of the trial (**Figures 4C, D**). Maximum anaphylaxis reaction score (mean control = 0, histamine = 3.4) and diarrhea status (mean control = 0, histamine = 0) were also recorded throughout the trial for the control and histamine mice as a secondary confirmation of anaphylaxis (**Figures 4E, F**). TEWL change at 15 min did not correlate with temperature change at 15 min (**Figure 4G**) but did correlate with anaphylaxis reaction score (**Figure 4H**). Serum was obtained via terminal bleed from mice following oral challenge and tested for MCPT-1 of the control (mean = 1,708.86 pg/mL) and histamine (mean = 1,468.61 pg/mL) mice (**Figure 4I**) and did not correlate with TEWL change at 15 min (**Figure 4J**), as expected.

### Passive systemic anaphylaxis

**Figure 5:**
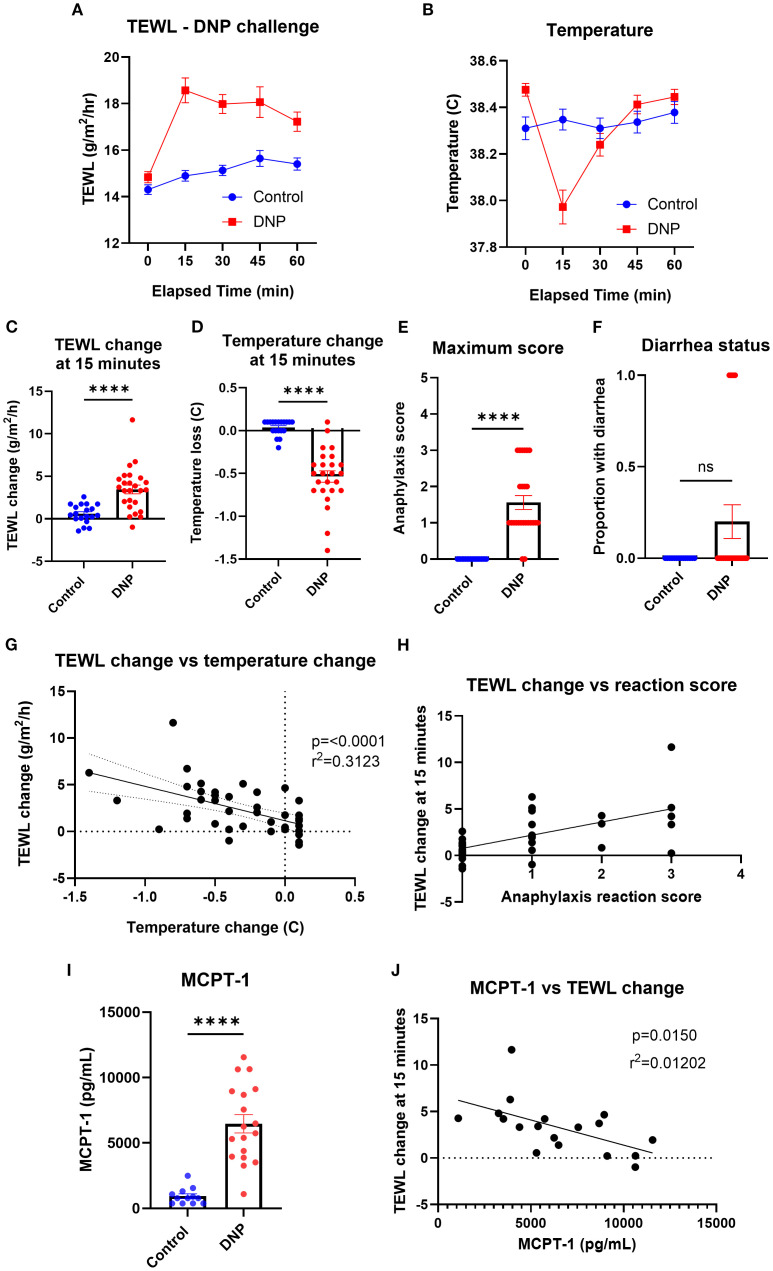
TEWL, temperature, anaphylaxis score, diarrhea status, and MCPT-1 measurements of control mice and mice orally challenged with DNP. **(A)** TEWL of mice taken at baseline and in 15-min intervals post-challenge for 60 min; Control *n* = 19, DNP *n* = 25. **(B)** Core temperature of mice taken at baseline and in 15-min intervals post-challenge for 60 min; Control *n* = 19, DNP *n* = 25. **(C)** TEWL of mice at 15 min subtracted from baseline TEWL to get TEWL change at 15 min. Control *n* = 19, DNP *n* = 25, unpaired *t*-test. **(D)** Core temperature of mice at 15 min subtracted from baseline TEWL to get TEWL change at 15 min. Control *n* = 19, DNP *n* = 25, unpaired *t*-test. **(E)** Highest score given to each mouse throughout the trial. Control *n* = 19, DNP *n* = 25, unpaired *t*-test. **(F)** Diarrhea status given to each mouse throughout the trial. Control (0) *n* = 19, DNP (0) *n* = 25 unpaired *t*-test. **(G)** Correlation of the mice’s TEWL change and their temperature change at 15 min. *n* = 44, simple linear regression. **(H)** Correlation of the mice’s TEWL change with their reaction score. *n* = 44, ordinary one-way ANOVA. **(I)** Interpolation of MCPT-1 concentration found in the serum of control and DNP mice. Control *n* = 12, DNP *n* = 18, unpaired *t*-test. **(J)** Interpolation of MCPT-1 concentration of murine serum correlated with TEWL change at 15 min; Control *n* = 12, DNP *n* = 18, linear regression fit shown. ****p<0.0001, ns, not significant.

To identify the impact of passive systemic anaphylaxis (PSA) on the TEWL of mice, TEWL and temperature were measured at baseline and in 15-min intervals post-oral challenge with DNP-challenged mice (**Figures 5A, B**). The TEWL and temperature change of DNP mice (mean TEWL change at 15 min = 3.48 g/m^2^/h; mean temperature change at 15 min = −0.55°C) changed the most at the 15-min time point, compared to that of the control mice (mean 0.91 g/m^2^/h; 0.03°C) (**Figures 5C, D**). Anaphylaxis reaction score (mean control = 0; DNP = 1.7) and diarrhea status were also recorded throughout the trial to corroborate the reaction status (**Figures 5E, F**). TEWL change (baseline TEWL subtracted from TEWL of mice at 15 min) correlated with temperature change (**Figure 5G**) as well as with anaphylaxis reaction score (**Figure 5H**). Serum MCPT-1 was obtained from control (mean = 1,210.83 pg/mL) and DNP (mean = 5,995.14 pg/mL) mice via terminal bleed following oral challenge (**Figure 5I**) and did not correlate with the TEWL change (**Figure 5J**).

### Active systemic anaphylaxis

**Figure 6:**
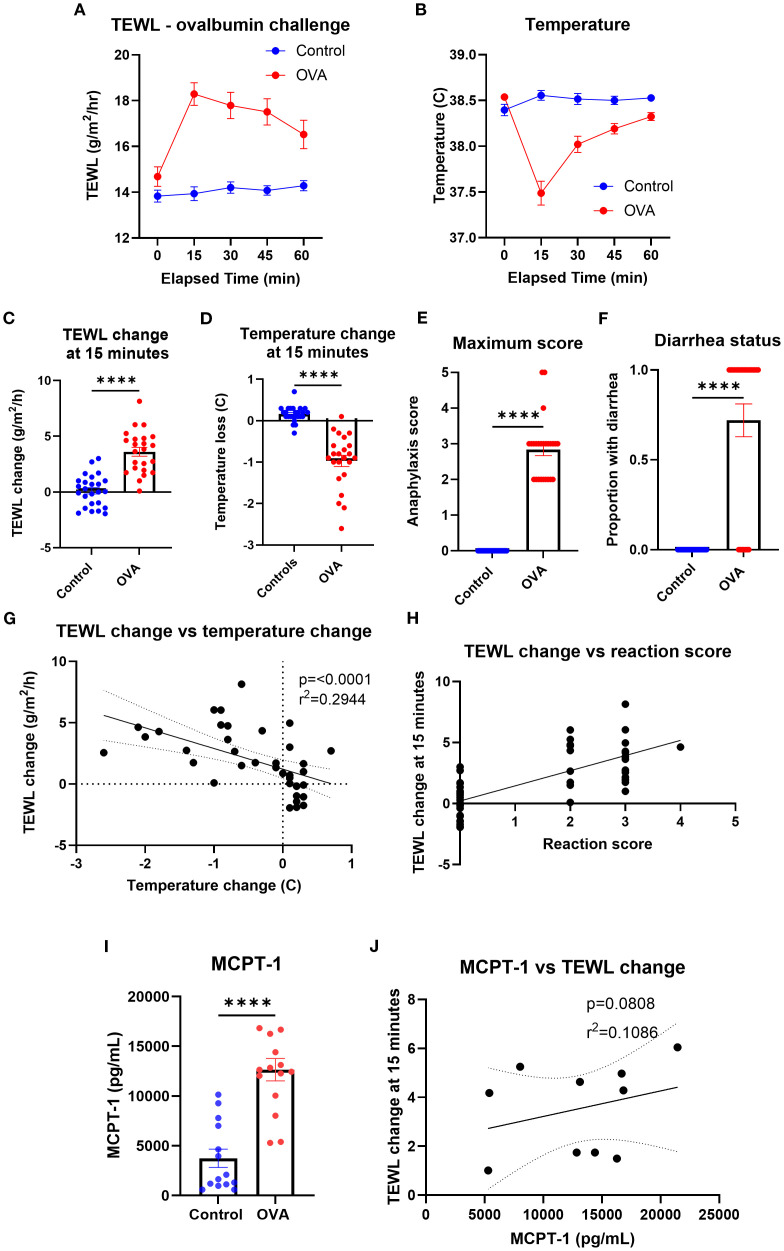
TEWL, temperature, anaphylaxis score, diarrhea status, and MCPT-1 measurements of control mice and mice orally challenged with OVA from trial 7. **(A)** TEWL of mice taken at baseline and in 15-min intervals post-challenge for 60 min; Control *n* = 25, OVA *n* = 23. **(B)** Core temperature of mice taken at baseline and in 15-min intervals post-challenge for 60 min; Control *n* = 25, OVA *n* = 23 **(C)** TEWL of mice at 15 min subtracted from baseline TEWL to get TEWL change at 15 min. Control *n* = 25, OVA *n* = 23, unpaired *t*-test. **(D)** Internal temperature of mice at 15 min subtracted from baseline TEWL to get TEWL change at 15 min. Control *n* = 25, OVA *n* = 23, unpaired *t*-test. **(E)** Highest score given to control and OVA mice throughout the trial. *n* = 15 per group, unpaired *t*-test. **(F)** Diarrhea status given to each control and OVA mice throughout the trial; Control *n* = 25, OVA *n* = 23, unpaired *t*-test. **(G)** Correlation of the mice’s TEWL change and their temperature change at 15 min. *n* = 48, simple linear regression. **(H)** Correlation of the mice’s TEWL change with their reaction score. *n* = 48, ordinary one-way ANOVA. **(I)** Interpolation of MCPT-1 concentration found in the serum of control and OVA mice. Control *n* = 14, OVA *n* = 15, unpaired *t*-test. **(J)** Interpolation of MCPT-1 concentration of murine serum correlated with TEWL change at 15 min; Control *n* = 14, OVA *n* = 15, linear regression line fit shown. ****p<0.0001, ns, not significant.

To identify the impact of ASA on the TEWL of mice, TEWL and temperature of OVA-sensitized mice were measured at baseline and in 15-min intervals post-oral challenge with OVA (only data from challenge number 7 used) (**Figures 6A, B**). The TEWL and temperature change of OVA-challenged mice (mean TEWL change at 15 min = 3.61 g/m^2^/h; mean temperature change at 15 min = −1.4°C) changed the most at the 15-min time point, compared to that of the control mice (mean TEWL change at 15 min = 0.11 g/m^2^/h; mean temperature change at 15 min = −0.97°C) (**Figures 6C, D**). Anaphylaxis reaction score and diarrhea status were also recorded for control (maximum anaphylaxis reaction score = 0; diarrhea status = 0) and OVA (mean maximum anaphylaxis reaction score = 2.84; diarrhea status = 0.72) mice throughout the trial to corroborate the reaction’s severity (**Figures 6E, F**). TEWL change at 15 min correlated with temperature change (**Figure 6G**) as well as with anaphylaxis reaction score (**Figure 6H**). Serum MCPT-1 was obtained via terminal bleed following oral challenge in control (mean = 3,738.46 pg/mL) and OVA mice (mean = 12,648.7 pg/mL) (**Figure 6I**), which trended toward a correlation with TEWL change (**Figure 6J**).

### Non-IgE-mediated vs. IgE-mediated models

**Figure 7:**
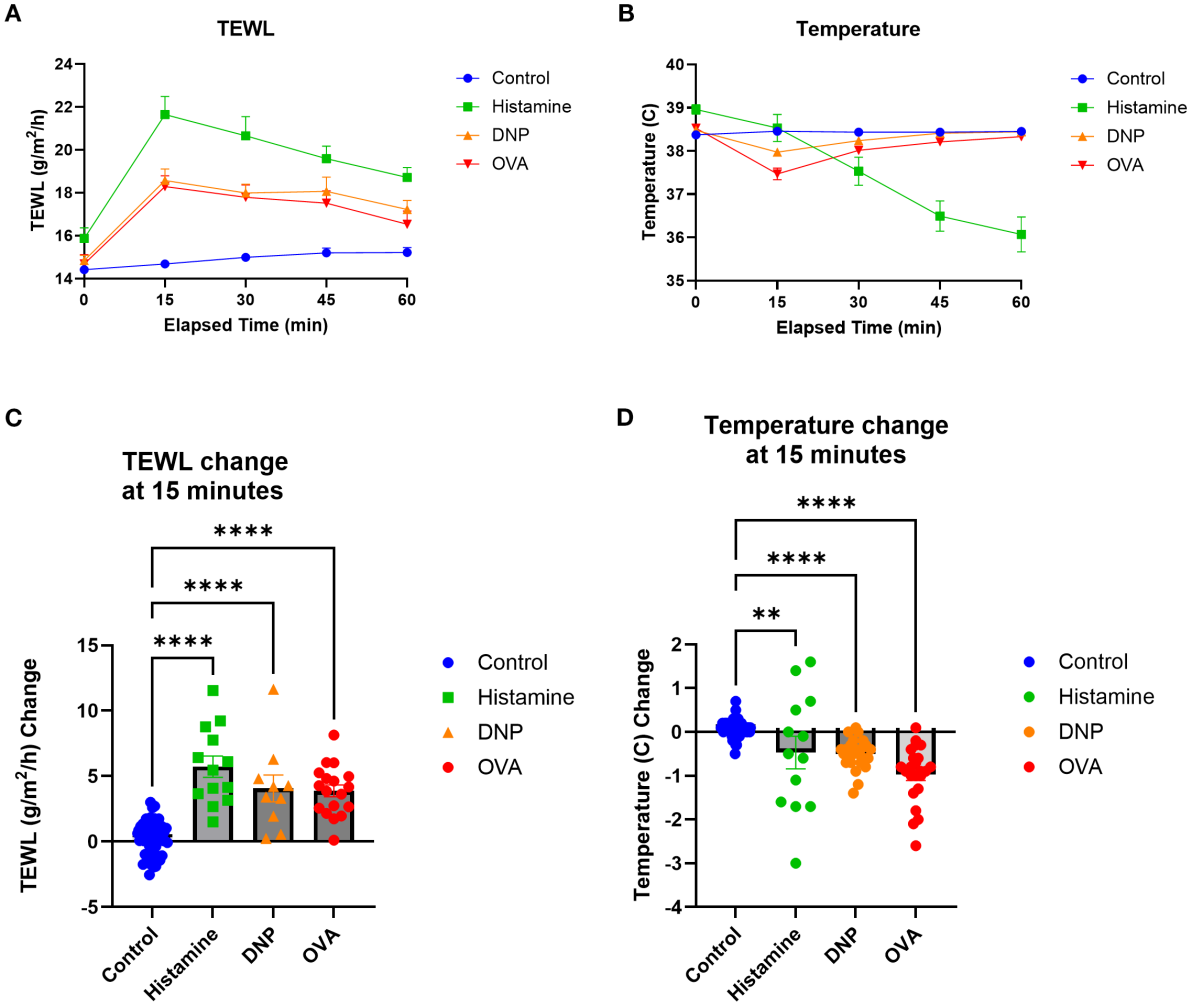
TEWL and temperature comparisons of the three models utilized. **(A)** TEWL of mice taken at baseline and in 15-min intervals post-challenge for 60 min. **(B)** Core temperature of mice taken at baseline and in 15-min intervals post-challenge for 60 min. **(C)** TEWL of mice at 15 min subtracted from baseline TEWL to get TEWL change at 15 min, one-way ANOVA. **(D)** Internal temperature of mice at 15 min subtracted from baseline TEWL to get TEWL change at 15 min, one-way ANOVA. Control *n* = 57, histamine *n* = 13, DNP *n* = 25, and OVA *n* = 23. ****p<0.0001, **p<0.001, ns, not significant.

To identify the impacts of varying types of anaphylaxes on TEWL measurements, histamine models (non-IgE-mediated) and DNP and OVA (IgE-mediated) models were compared by TEWL results (**Figure 7A**), TEWL change at 15 min (**Figure 7B**), temperature (**Figure 7C**), and temperature change at 15 min (**Figure 7D**). TEWL increased significantly for all models, whereas other markers such as anaphylaxis reaction severity and temperature change varied greatly depending on which model was utilized. In addition, in order to evaluate the total change in TEWL during the entire challenge time course, we calculated the AUC for TEWL in each challenge. We then correlated the values with temperature change at 15 min, maximum anaphylaxis severity score, and MCPT-1 results (**Supplementary Figure S1**). To determine the effect of anaphylaxis on TEWL during earlier challenges, prior to the seventh trial, TEWL change at 15 min, temperature change at 15 min, maximum anaphylaxis severity score, and diarrhea status were recorded and plotted from Trial 4 through Trial 6 (**Supplementary Figure S2**).

## Discussion

FA persists as a profound societal health issue with nearly 8% of children and 10% of adults affected in the United States (15, 16). Food anaphylaxis, an unpredictable, potentially deadly consequence of FA, causes 200,000 annual US emergency room visits (1–3). The OFC remains the gold standard diagnostic test for FA, but OFCs carry anaphylaxis risk, especially for those in clinical trials expecting a reaction on entry OFC (6, 7, 12). Unfortunately, the anaphylaxis endpoint relies purely on a clinical diagnosis through physician observation, and no approved monitoring device is available (9, 13). However, early identification and treatment of anaphylaxis can reduce reaction severity and minimize adverse outcomes (14). Our group has previously shown that TEWL increases during human FA reactions and likely provides some level of advanced warning for anaphylaxis (4, 5, 8).

TEWL is a well-established measure of skin barrier function and is used in evaluating topical medications and in dermatological conditions (17). TEWL is measured painlessly and noninvasively using skin contact probes to give real-time results, and newer technology allows for continuous measurement over several hours (15, 16). Presently, the only objective measurement in mice to confirm anaphylaxis in real time is invasive rectal temperature measurement, which does not correlate fully with what occurs in human food anaphylaxis pathophysiology (23). Core temperature measurement in mice may not consistently correlate with other anaphylaxis measures in all models, such as symptom scores or MCPT-1 results (25–27), supporting the need for an additional objective reaction correlate, such as TEWL. TEWL has never been reported in mouse models of food anaphylaxis, so in this study, we sought to evaluate whether TEWL could provide a similar measure of cutaneous change in such models as in human food anaphylaxis in order to predict the onset of anaphylaxis. This could facilitate a bedside–bench–bedside virtuous cycle to facilitate a better understanding of anaphylaxis pathophysiology. Of particular interest in this study, we find that TEWL increased in a comparable manner in all three reaction models, and preceded the temperature drop in a histamine model. This suggests that TEWL represents a shared outcome regardless of allergic stimulus that may be useful in standardizing allergic models.

Prior studies with TEWL in mouse models provide variable or limited technical information on effective device use or measurement, and no reviews are available to describe a standardized measurement approach (18–22). There are a variety of effective mouse models of FA and anaphylaxis (23, 28). We sought to define a standardized TEWL measurement approach for food anaphylaxis modeling to support future research into food anaphylaxis mechanisms. We note that the ear is a commonly targeted site in mice for skin evaluations (29, 30) and for various anaphylaxis models, particularly for vascular effects of allergic reactions (31, 32). Thus, optimizing TEWL to the mouse ear provides a useful correlation to well-established models commonly used in the food anaphylaxis space.

While a great deal of detail on the mechanisms of food anaphylaxis is known (33–35), key specifics around how the skin dynamically changes during anaphylaxis remain understudied. One mechanism of the TEWL change in food anaphylaxis may relate the effect of histamine on the vasculature in anaphylaxis (36). We demonstrate that direct IV injection of high-dose histamine, sufficient to replicate anaphylaxis symptoms, readily induces a TEWL increase without evidence of underlying MC activation and with a slower onset of temperature loss. Anaphylaxis is associated with intense peripheral vasodilation (31, 32). Histamine is known to have a direct effect on vascular endothelial integrity, leading to interstitial leaking of fluid (37). Furthermore, in human food anaphylaxis, serum albumin decreases during more intense anaphylaxis, suggesting a high level of extravasation with worse reactions (38). Vascular leak has long been associated with the observation of temperature loss during anaphylaxis in the mouse (39), so the lack of a temporal association between histamine-driven TEWL increase and a slower temperature loss suggests that additional factors may be at play. For example, mast cell granule contents can directly antagonize cell adhesion molecules; if there is an acute cutaneous release of MC granules, this could directly affect the skin barrier in real time as an alternate or additive mechanism for the increase seen in TEWL (38). To this point, we see a trend toward a correlation between the ASA model’s MCPT-1 release and the TEWL increase, suggesting that a more “complete” immune response driving the reaction may include just such a mechanism. Altogether, the observations here suggest that more is occurring at or just below the skin during food anaphylaxis than is fully understood, supporting the need for additional investigations into the role of cutaneous changes in anaphylaxis pathogenesis.

This study has several limitations. First, this study does not directly assess the mechanism of the TEWL changes observed. In addition, not all food anaphylaxis models were utilized, such as cholera toxin or skin sensitization models, and non-anaphylaxis-producing models were not included either. A largely method-focused approach was taken to provide a clear framework for future mechanistic work that will follow and to provide the field with details on TEWL use in food anaphylaxis models more promptly. In this context, additional food anaphylaxis models can utilize the TEWL framework provided here. Furthermore, this study focuses on non-anesthetized mice with ear-focused TEWL measurements, while other methods might require anesthesia or cutaneous barrier measurements on other areas of the body. As we show in the baseline measurements, other body areas may present challenges for TEWL measurement, and so that work may need additional optimization.

To summarize, we show that TEWL increases significantly and rapidly in multiple food anaphylaxis models in mice, even when no temperature drop had been observed. This supports the potential of TEWL as a noninvasive predictor to anaphylaxis for multiple stimulation approaches. This work demonstrates that TEWL can be readily measured in mouse models of food anaphylaxis regardless of stimulus approach with reproducible and largely stable baseline values. Furthermore, TEWL rises during mouse food anaphylaxis in a manner akin to human food anaphylaxis, which will facilitate mechanistic investigations into the rapid cutaneous changes during the early phases of food anaphylaxis.

## Data Availability

Any data requests for this manuscript require review and approval by the University of Michigan Office for Research and Sponsored Programs. Requests to access the datasets should be directed to CS, schulerc@med.umich.edu.
