# Supplementary material for: Transepidermal water loss increases during murine food anaphylaxis and reflects reaction severity
Source: Front Immunol. 2025 Oct 2;16:1667569. doi: 10.3389/fimmu.2025.1667569 (PMC12528144; doi:10.3389/fimmu.2025.1667569)
Supplement: Supplementary file 1 [file SupplementaryFile1.docx]

**
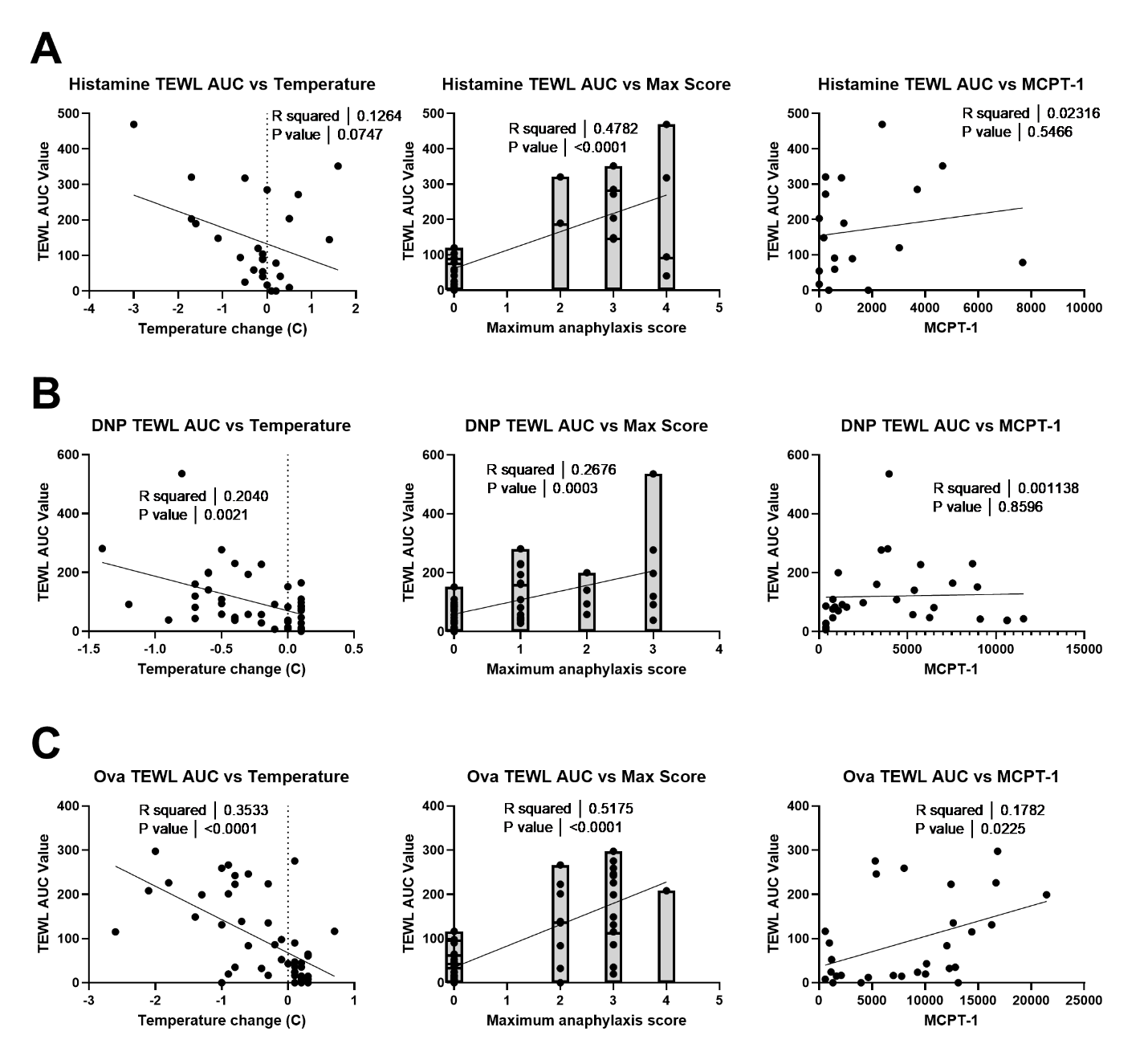
**

**Supplemental Figure 1: Area under the curve transepidermal water loss data analysis.** In each case, the TEWL results were converted to a total area under the curve (AUC) value for each mouse’s data. This was then paired in an XY graphical comparison for temperature change at 15 minutes, maximum anaphylaxis score, and MCPT-1 results. Then, a simple linear regression was fitted to each dataset, and the R squared and P values reported in each sub-panel are listed for each specific linear regression model. Results are reported for all three measures for the (A) Histamine, (B) DNP, and (C) ovalbumin models. TEWL = transepidermal water loss, AUC = area under the curve, DNP = dinitrophenol, ova = ovalbumin.


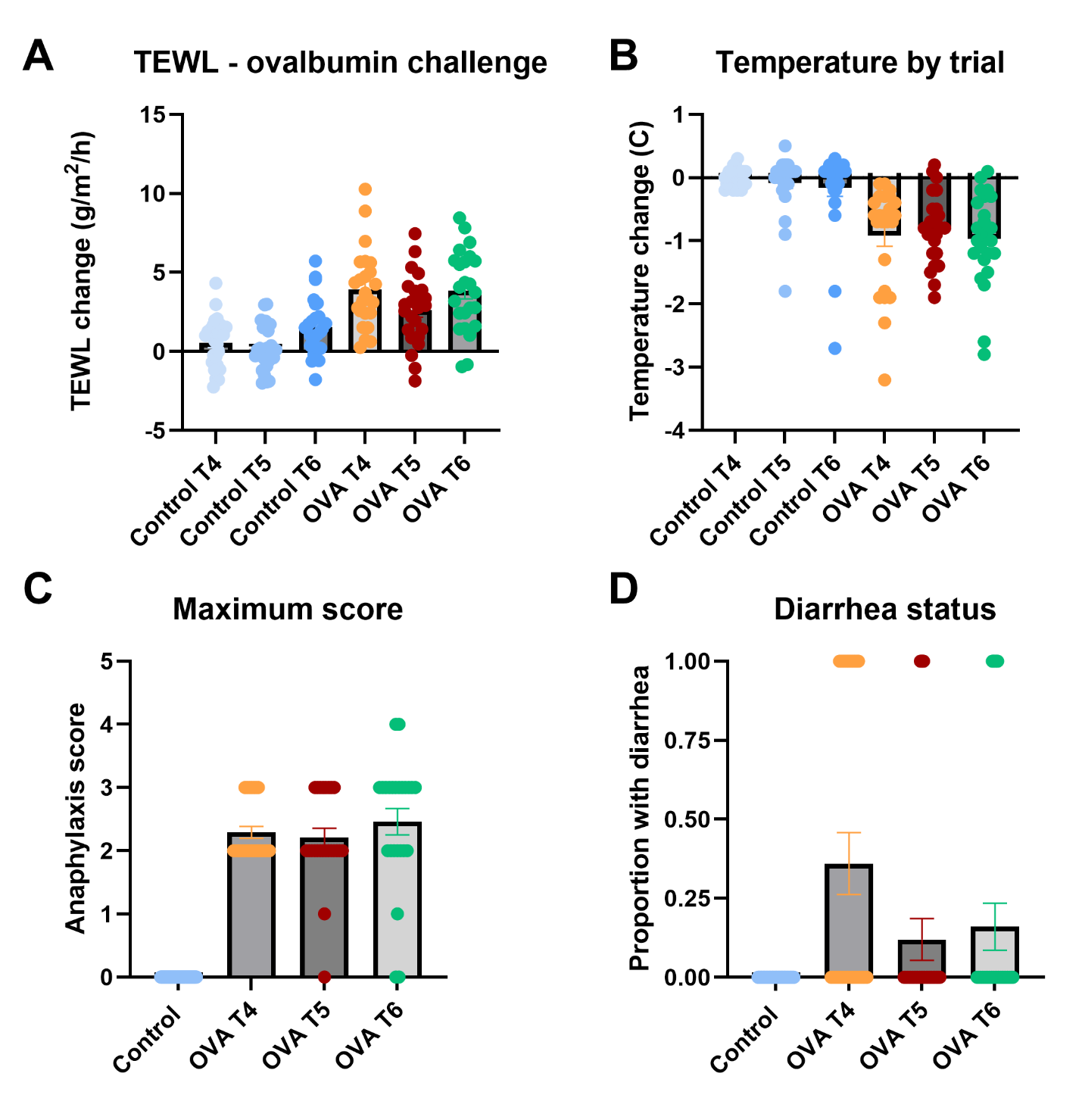


**Supplemental Figure 2: TEWL, temperature, anaphylaxis score, and diarrhea status measurements of control mice and mice orally challenged with OVA from trials 4-6.** (A) From trials 4 through 6, TEWL of control mice and mice orally challenged with OVA at 15 minutes subtracted from baseline TEWL to get TEWL change at 15 minutes. Control n = 25 each, OVA n = 24 each, ordinary one-way ANOVA. (B) Internal temperature of mice at 15 minutes subtracted from baseline TEWL to get TEWL change at 15 minutes. Control n = 25, OVA n = 24, ordinary one-way ANOVA. (C) Highest score given to control and OVA mice for trials 4, 5, and 6. Control n = 25, OVA n = 24 for each group, ordinary one-way ANOVA. (D) Diarrhea status given to each control and OVA mice during trials 4, 5, and 6. Control n = 25, OVA n = 24 for each group, ordinary one-way ANOVA.
